# Optimal Task-Dependent Changes of Bimanual Feedback Control and Adaptation

**DOI:** 10.1016/j.cub.2007.08.051

**Published:** 2007-10-09

**Authors:** Jörn Diedrichsen

**Affiliations:** 1 Wolfson Centre for Cognitive Neuroscience, School of Psychology, University of Wales, Bangor, Gwynedd LL57 2AS, United Kingdom

**Keywords:** SYSNEURO

## Abstract

The control and adaptation of bimanual movements is often considered to be a function of a fixed set of mechanisms [1, 2]. Here, I show that both feedback control and adaptation change optimally with task goals. Participants reached with two hands to two separate spatial targets (two-cursor condition) or used the same bimanual movements to move a cursor presented at the spatial average location of the two hands to a single target (one-cursor condition). A force field was randomly applied to one of the hands. In the two-cursor condition, online corrections occurred only on the perturbed hand, whereas the other movement was controlled independently. In the one-cursor condition, online correction could be detected on both hands as early as 190 ms after the start. These changes can be shown to be optimal in respect to a simple task-dependent cost function [3]. Adaptation, the influence of a perturbation onto the next movement, also depended on task goals. In the two-cursor condition, only the perturbed hand adapted to a force perturbation [2], whereas in the one-cursor condition, both hands adapted. These findings demonstrate that the central nervous system changes bimanual feedback control and adaptation optimally according to the current task requirements.

## Results and Discussion

**Figure 1:**
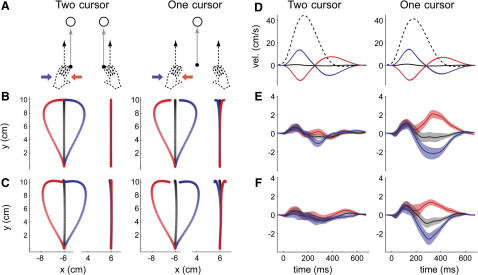
Experiment 1 Shows Bilateral Movement Corrections in the One-Cursor Condition (A) In the two-cursor condition, participants reached for two separate targets. In the one-cursor condition, they reached with both hands to move a common cursor to a single target. One of the hands was perturbed with a leftward (red) or rightward (blue) force field or was unperturbed (black). (B) Predicted movement trajectories based on the optimal control policy. (C) Movement trajectories observed in experiment 1, averaged across participants and hands. (D) The y velocity (dashed line) and x velocity (red, blue, and black solid lines) of the perturbed hand. (E and F) The x velocity of the unperturbed hand with (E) and without (F) visual feedback shows the onset of the correction in the one-cursor condition. The shaded area indicates the across-subject standard error (SE).

Optimal control theory [3] predicts that the coordination of movements depends on task goals. Here, I show that humans alter bimanual feedback control according to this prediction. In experiment 1, participants (n = 10) performed a bimanual reaching task in separate sessions under two conditions (Figure 1A). In the two-cursor condition, participants simultaneously moved two cursors, one with each hand, toward separate visual targets. In the one-cursor condition, participants performed physically similar reaching movements but moved a single cursor, presented at the average position of the two hands, to a single target. So that feedback control could be tested, in half of the trials, a randomly selected hand was perturbed with a velocity-dependent leftward or rightward force field [4].

The optimal feedback control policy for each condition, i.e., the mapping from the state of the two hands to motor commands, can be derived by the minimization of a task-dependent cost function. For the two-cursor condition, the cost function penalizes the distance between of the left and right hands ($p_{L}$and $p_{R}$) from their respective targets (*g*), and any residual velocity (v) in the end of movement, plus the total sum of the squared motor commands (*u*):

(1) $$J=\sum_{t=1}^{T}w_{p,t}\left({\left\|p_{L,t}-g_{L}\right\|}^{2}+{\left\|p_{R,t}-g_{R}\right\|}^{2}\right)+w_{v,t}\left(v_{L,t}^{2}+v_{R,t}^{2}\right)+w_{u}\left(u_{L,t}^{2}+u_{R,t}^{2}\right).$$

By using a plausible model of the human arm, I derived the weights of the cost function to fit the velocity profile of unperturbed movements (see the Experimental Procedures for details). Furthermore, I estimated arm stiffness to fit the movement of the perturbed hand in the two-cursor condition. I then asked what movements would be predicted for the unperturbed hand. The optimal control policy for the two-cursor task controls the left and right hands independently; when one hand is perturbed by a velocity-dependent force field, corrections are only performed with that hand (Figure 1B, left). This prediction was confirmed by the experimental data. No significant changes in the movement of the unperturbed hand were found (Figures 1C–1E, left).

For the one-cursor condition, I changed the spatial term to reflect the distance between the cursor and target, rather than between individual hands and targets:

(2) $$J=\sum_{t=1}^{T}w_{p,t}{\left\|\frac{x_{L,t}+x_{R,t}}{2}-g\right\|}^{2}+...$$

All other system and cost function parameters were kept the same. Under this cost function, the system produces the same unperturbed movements as in the two-cursor condition; however, it responds very differently to perturbations. When one hand is perturbed, both hands make corrections, minimizing the overall motor commands necessary (Figure 1B, right).

**Figure 2:**
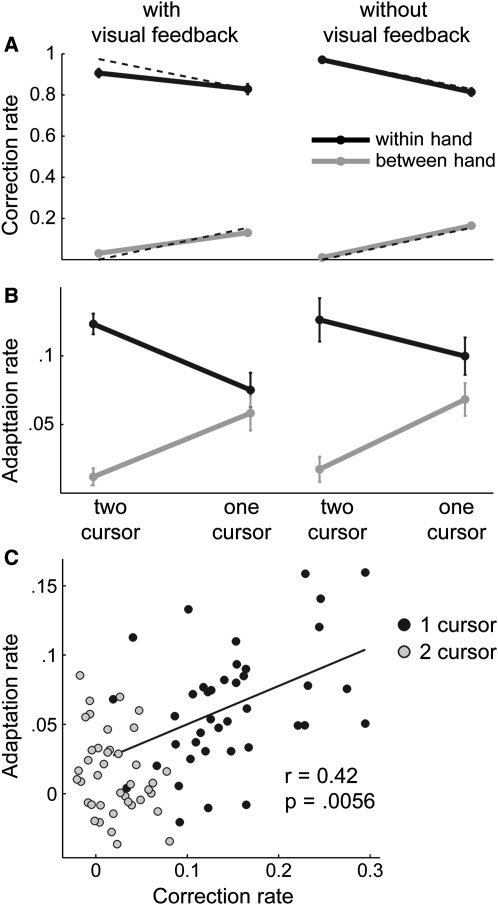
Task-Dependent Changes in Correction and Adaptation Rates (A) Correction rate, the proportion of initial direction error corrected by the same (black) and other (gray) hand within the same trial. The predicted optimal correction rates are plotted as dotted lines. (B) Adaptation rate, the influence of an initial direction error onto the initial direction of the same (black) and other (gray) hand. Error bars indicate the between-subject standard error of the mean (SEM). (C) For the one-cursor condition only, the between-hand correction rate correlates significantly with the between-hand adaptation rate. Each data point represents one hand of one participant under either the visual-feedback or no-visual-feedback condition.

The behavior of the participants followed closely this prediction (Figures 1C–1E, right). The kinematics of the movements during unperturbed movements was similar. However, starting at 190 ms, the unperturbed hand showed coordinated corrections; at this time, the lateral velocity started to depend significantly on the direction of the force applied to the other hand. As a quantitative measure of the amount of correction, I computed the proportion of the initial direction error corrected by each hand (Figure 2A). When going from the two- to the one-cursor condition, the between-hand correction rate increased, whereas the within-hand correction rate decreased, matching quantitatively the predictions of optimal control theory.

This finding might simply reflect the visual feedback; just seeing the common cursor being perturbed is enough to elicit bilateral corrections. So that this could be tested, the visual feedback of the cursor was withdrawn in half of the trials, making the one- and two-cursor conditions identical except for task instructions. Even without visual feedback, participants showed similar bilateral reactions in the one-cursor condition (Figures 1F and 2A). Thus, just by changing whether participants thought they controlled one or two end effectors, feedback control based on proprioceptive information could be manipulated.

**Figure 3:**
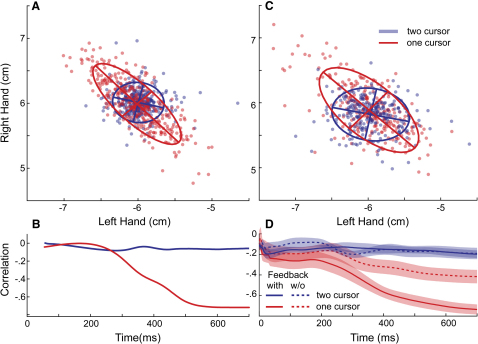
Endpoint Correlation in Unperturbed Movements (A) Predicted endpoint correlation in the x direction in the two- (blue) and one- (red) cursor condition, with the same simulation parameters as in Figure 1. (B) Predicted time course of the correlation between movement directions. (C) Endpoint correlation of one representative participant in experiment 1. (D) Time course of correlation, averaged across all participants of experiment 1, with the shaded area indicating the SEM.

When adding signal-dependent noise separately to the movement of each hand, the optimal control policy for the one-cursor condition predicts that the endpoints of the two hands should become negatively correlated. The effect arises because of bilateral corrections of motor noise, and should gradually arise over the course of the movement (Figures 3A and 3B). Congruent with this prediction, the movement endpoints on unperturbed trials were more negatively correlated in the one-cursor than in the two-cursor condition, both with (−0.81 versus −0.22, t(9) = 7.283, p < 0.001) and without (−0.44 versus −0.18, t(9) = 3.881, p = 0.004) visual feedback. Furthermore, the effect arose after the predicted time course (Figures 3C and 3D). Thus, participants corrected only for task-relevant error [3, 5], whereas negative covariation of the hands accumulated.

I then investigated how participants adapted to the force fields. Previous studies have found the independent adaptation of each arm during bimanual movements [2]. By using a state-space model (see the Experimental Procedures), I estimated how much the initial direction of each hand in trial n + 1 (at 160 ms) was influenced by a perturbation experienced in trial n, either on the same hand (within-hand adaptation rate) or on the other hand (between-hand adaptation rate).

In the two-cursor condition, the within-hand adaptation rate was 0.12, with limited generalization to the other hand (Figure 2B). In the one-cursor condition, the between-hand adaptation rate was significantly higher [t(9) = 3.37, p = 0.008] and the within-hand significantly lower [t(9) = 2.43, p = 0.037]. Here, a force field applied to one hand changed the initial direction in both hands. Thus, how participants adapted to a unilateral force field changed substantially with the task goals. A possible account for the parallel changes feedback control and adaptation is that the correction of each hand in the last trial dictates how much the movement should adapt in the next [6]. Consistent with this hypothesis, the between-hand correction and adaptation rate in experiment 1 were significantly correlated (Figure 2C).

**Figure 4:**
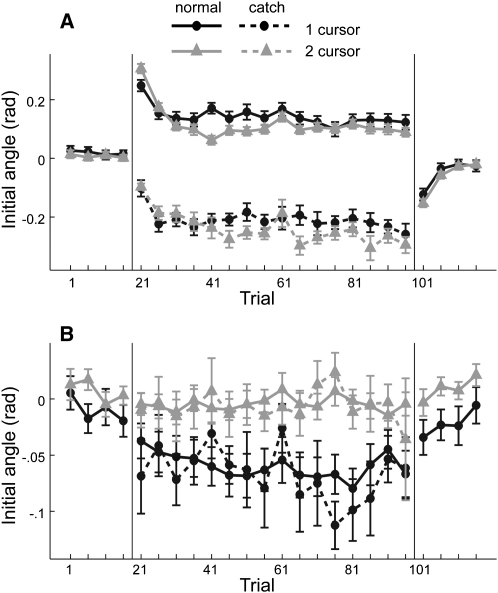
Experiment 2 Shows Bilateral Adaptation to a Constant Force Field in the One-Cursor Condition The initial direction error of the perturbed hand (A) and unperturbed hand (B) in normal (solid line) and catch (dashed line) trials. Results are averaged across participants, hands, and force fields. Error bars indicate the between-subject standard error of the mean (SEM).

So that the changes in adaptation could be confirmed, participants in experiment 2 (n = 8) adapted for 80 trials to a velocity-dependent force field of a constant direction applied to one of the hands. The perturbed hand (Figure 4A) quickly adapted to the large initial errors. This can also be seen in the change of initial direction in catch trials, in which no force field was applied (dashed line). In the two-cursor condition, adaptation was restricted to the perturbed hand. In the one-cursor condition, however, the unperturbed hand (Figure 4B) also changed its initial direction, such that it opposed the force field [t(7) = 7.89, p < .001], allowing the perturbed hand to adapt less than in the two-cursor condition [t(7) = 2.67, p = 0.032].

Previous work has shown that the control of bimanual movements can change with task requirements [5, 7], visual feedback [8, 9], and attention [10]. Here, I provide another clean demonstration of this important feature of the human motor system and show that this flexibility can be well described as the optimization of simple task-dependent cost functions. The predictions of optimal control theory are qualitatively robust over a wide range of parameter values and are well matched by the empirical data. The results also show that changes in optimal feedback control influence how the motor system adapts to novel environments.

## Experimental Procedures

### Experiment 1 Methods

All experimental procedures were approved by the ethics committee of the School of Psychology at the University of Wales, Bangor. Ten right-handed participants (two of them male, average age = 21.3 years) made 10 cm reaching movements while holding on to a robotic device with each hand (Phantom 3.0, SensAble Technologies). Movements were performed in the natural reaching space in an upward-forward direction, involving shoulder and elbow movements, with the elbow pointing downwards. A simulated spring (150 N/m) restricted the movements to a frontoparallel plane, 20° from vertical. A horizontal crossbar stabilized the upper body and minimized interaction torques between left and right arm movements. By using two mirrors mounted at 90° to each other, participants viewed one monitor with the left eye and one monitor with the right. This stereoscopic display was calibrated to the robotic devices such that stimuli could be displayed at their veridical 3D locations.

Participants moved the two cursors (8 mm diameter) into the starting spheres, displayed 6 cm to the left and right of the body midline at breast height. In the two-cursor condition, two targets (8 mm diameter) were presented 10 cm in a forward-upward direction above the starting spheres. In the one-cursor condition, a single target was presented at body midline, and a single cursor was presented at the spatial average position of the two hands. Participants were instructed to reach the target(s) by moving both hands rapidly upwards. A movement started when the hand reached a speed of 3 cm/s and ended when the speed fell below 1.5 cm/s for 30 ms. Movement times of less than 700 ms with a spatial accuracy of better than 5 mm were rewarded.

Participants performed one session in the one-cursor condition and one session in the two-cursor condition. Sessions were separated by at least one day, and their order was counterbalanced between participants. Each session consisted of nine blocks of 64 trials. The first block was a training block with only unperturbed trials, and eight blocks with random perturbations followed: In half of the trials, a leftward or rightward force field was presented to one of the hands with equal probability. The sideways force was proportional (3.5 Ns/m) to the upward velocity along the movement plane. For the last six blocks of each session, the visual feedback of the cursor was withdrawn between the start and end of the movement on half the trials. The trials were randomly chosen, such that participants did not know until after the movement started whether they would have visual feedback or not.

### Experiment 2 Methods

Eight different right-handed participants (one1 of them male, average age = 21) were run in experiment 2. The apparatus, movements, and stimuli were identical to those used in experiment 1. The experiment consisted two practice blocks of 60 trials, the first in the two-cursor condition, and the second in the one-cursor condition. The participants then performed eight adaptation runs, each consisting of 20 unperturbed movements, 80 movements with a velocity-dependent force field applied to one of the hands, and 20 unperturbed movements. The force field was identical to the one used in experiment 1, but had a constant direction for the whole adaptation run. Twelve out of the 80 movements were randomly chosen to be catch trials in which no force was present. Each participant performed one adaptation run for each combination of force-field direction (leftward versus rightward), perturbed hand (left versus right), and condition (one cursor versus two cursor). The sequence of runs was randomly determined for each participant and distributed across two 1 hr sessions, separated by at least 1 day.

### Data Analysis

The initial direction error (*y*) error of each movement was defined as the angular deviation from a straight-ahead movement at 160 ms after the movement start. The online correction (*c*) was the difference between the direction of the overall movement and the initial movement direction. The correction rates, the proportion of error corrected by each hand, were calculated with a linear regression model of the online correction of the left and right hands as a function of the initial direction error of each hand in that trial:

(3) $${\left[\begin{array}{c} c_{L}\\ c_{R} \end{array}\right]}_{n}=\left[\begin{array}{cc} C_{L} & C_{LR}\\ C_{RL} & C_{R} \end{array}\right]{\left[\begin{array}{c} y_{L}\\ y_{R} \end{array}\right]}_{n}.$$

$C_{L}$and $C_{R}$are the within-hand correction rates, and $C_{LR}$and $C_{RL}$are the between-hand correction rates.

In experiment 1, random force-field perturbations were applied. To model trial-by-trial adaptation, I used a state-space approach [11], in which the initial direction of the left and the right hand (*y*) was expressed as a function of the force applied to the hand (*a*) and the internal state of the system (*z*),

(4) $${\left[\begin{array}{c} y_{L}\\ y_{R} \end{array}\right]}_{n}=\left[\begin{array}{cc} D_{L} & 0\\ 0 & D_{R} \end{array}\right]{\left[\begin{array}{c} a_{L}\\ a_{R} \end{array}\right]}_{n}-{\left[\begin{array}{c} z_{L}\\ z_{R} \end{array}\right]}_{n},$$

in which $D_{L}$and $D_{R}$ relate to the stiffness of the left and right arm. Learning was modeled as a change in internal state from trial n to trial n + 1 as a function of the initial direction error in trial n,

(5) $${\left[\begin{array}{c} z_{L}\\ z_{R} \end{array}\right]}_{n+1}={\left[\begin{array}{c} z_{L}\\ z_{R} \end{array}\right]}_{n}+\left[\begin{array}{cc} B_{L} & B_{LR}\\ B_{RL} & B_{R} \end{array}\right]{\left[\begin{array}{c} y_{L}\\ y_{R} \end{array}\right]}_{n},$$

in which $B_{L}$and $B_{R}$ are the within-hand adaptation rates, and $B_{LR}$ and $B_{RL}$ are the between-hand adaptation rates. For each participant and condition, the maximum-likelihood estimates of the parameters were found by the minimization of the sum of squares between observed and predicted initial direction errors. I estimated separate B parameters depending on whether trial n had visual feedback.

### Optimal Control Simulations

So that the predictions of optimal feedback control could be calculated, a biologically inspired model of a two-degree-of-freedom arm [4] was used. Most parameters were kept identical to previous work [12]. For simplicity, I used a local linear approximation of the system in Euclidian coordinates. However, very similar results can be obtained with a nonlinear, joint-based model, by using iterative techniques to derive the optimal control policy [13]. In our model, each hand is conceptualized by a state vector that contains the x and y component in Euclidian coordinates of position (p), velocity (v), force (f), muscle activation (h), and target position (g). The kinematics are modeled with

(6) $$\begin{array}{l} p_{t+1}=p_{t}+\Delta tv_{t}\\ v_{t+1}=v_{t}+\Delta t\frac{f_{t}}{I} \end{array},$$

in which Δt is the size of the discrete time step in ms, *t* is the number of time steps, and *I* is the inertia of the arm at the endpoint (0.5kg). A stiffness term was added in the x direction for the perturbed hand and fitted to the response of the perturbed hand to the force-field perturbation in the two-cursor condition, resulting in an estimate of 129.9 N/m. I simulated the delay in the muscles by using two-coupled first-order low-pass filters with time constants $\tau_{1}$ = $\tau_{2}$ = 40 ms. Therefore, force (*f*) was a low-pass-filtered version of the motor command *u*:

(7) $$\begin{array}{l} f_{t+1}=f_{t}+\Delta t/\tau_{2}\left(h_{t}-f_{t}\right)\\ h_{t+1}=h_{t}+\Delta t/\tau_{1}\left(u_{t}-h_{t}\right) \end{array}.$$

To simulate sensory delay, I expanded the state space with a series of four-coupled first-order filters for the sensed position, velocity, and force:

(8) $$x_{t+1}^{\left(j\right)}=x_{t}^{\left(j\right)}+\Delta t/\tau_{s}\left(x_{t}^{\left(j-1\right)}-x_{t}^{\left(j\right)}\right),\text{with}j=1,\ldots ,4.$$

The current state of the system is $x^{\left(1\right)}$, and the state that can be sensed is $x^{\left(4\right)}$, in which *x* is used as a placeholder for *p*, *v*, or *f*. The time constant for each filter was $\tau_{s}$ = 15 ms. In summary, the state vector for each hand is

(9) $$x_{t}={\left[\begin{array}{cccccccccccc} p_{t}^{\left(1\right)} & v_{t}^{\left(1\right)} & f_{t}^{\left(1\right)} & h_{t} & g & p_{t}^{\left(2\right)} & v_{t}^{\left(2\right)} & f_{t}^{\left(2\right)} & ... & p_{t}^{\left(4\right)} & v_{t}^{\left(4\right)} & f_{t}^{\left(4\right)} \end{array}\right]}^{T},$$

and the sensed state is

(10) $$y_{t}=\left[\begin{array}{ccc} p_{t}^{\left(4\right)} & v_{t}^{\left(4\right)} & f_{t}^{\left(4\right)} \end{array}\right].$$

By defining the appropriate matrices, whole time-discrete system can be written as

(11) $$\begin{array}{l} {\left[\begin{array}{c} x_{L}\\ x_{R} \end{array}\right]}_{t+1}=A{\left[\begin{array}{c} x_{L}\\ x_{R} \end{array}\right]}_{t}+B\left[\begin{array}{c} u_{L}\\ u_{R} \end{array}\right]\\ {\left[\begin{array}{c} y_{L}\\ y_{R} \end{array}\right]}_{t+1}=H{\left[\begin{array}{c} x_{L}\\ x_{R} \end{array}\right]}_{t} \end{array}.$$

It is important to note that A, B, and H are block diagonal; i.e., in the system dynamics, the left hand has no influence on the right and vice versa.

The position and velocity term in the cost function of the two-cursor reaching (Equation 1) increased exponentially during the movement up to the maximal movement time (900 ms), with time constant of $\tau_{\text{cost}}$ = 51 ms:

(12) $$\begin{array}{l} w_{p,t}=c_{p}\exp\left(-\frac{\left(MT-t\Delta t\right)}{\tau_{\text{cost}}}\right)\\ w_{v,t}=c_{v}\exp\left(-\frac{\left(MT-t\Delta t\right)}{\tau_{\text{cost}}}\right) \end{array}.$$

An exponential increase in goal cost allows us to treat the movement time as a soft rather than a hard constraint for human movement production [14]. $c_{p}$and $c_{v}$were chosen such that the sum of the respective weights over the whole movement were 40 1/m^2^ and 5 s^2^/m^2^. The weight for the control cost summed to 5^∗^10e-5 over 1 s. The parameters of the cost function were estimated to provide the best fit to the velocity profile of unperturbed movements. For the one-cursor task, I used the same cost function but replaced the position term with a common term (Equation 2).

By using the cost function, I calculated the optimal control gains $L_{t}$ with the Riccati equations (see the Supplemental Data available online) [15]. The control gains allow us to compute the optimal motor commands given an estimate of the current state, $u_{t}=-L_{t}{\hat{x}}_{t}^{}$. For the two-cursor task, all $L_{t}$ are block-diagonal matrices; i.e., control of the two hands is independent. For the one-cursor task, the optimal control gains $L_{t}$ have nonzero off-diagonal entries; i.e., control of the left hand depends on the estimated state of the right hand and vice versa.

For the simulations in Figure 1, the system was perturbed with the force fields from experiment 1. No noise was added to the motor output, and sensory noise was assumed to be of the same size as used in [12]. For the simulations in Figure 3, I used the same system and control policies as in Figure 1, but I added signal-dependent noise to the control commands in vector *u* [12]. The standard deviation of the noise depended linearly on the size of control signal. Because the simulation was Euclidian rather than in joint space, the noise in each direction was made dependent on linear combination of the motor commands in the x and y directions, such that the resulting spatial noise variances within each hand in the two-cursor condition were matched to the experimental data. With these parameters, I then simulated the one-cursor condition. A more detailed description of the model, and an implementation in Matlab (Natick, MA), is provided in the Supplemental Data.

## Supplemental Data

Document S1. Experimental Procedures and Three Figures

Document S2. Optimal Control Model for Bimanual Reaching Task
