# Supplementary material for: Optimal Task-Dependent Changes of Bimanual Feedback Control and Adaptation
Source: Curr Biol. 2007 Oct 9;17(19):1675–9. doi: 10.1016/j.cub.2007.08.051 (PMC2230536; doi:10.1016/j.cub.2007.08.051)
Supplement: Document S1. Experimental Procedures and Three Figures [file mmc1.pdf]

Supplemental Data

Optimal Task-Dependent Changes of Bimanual Feedback Control and Adaptation

Jörn Diedrichsen

Supplemental Discussion

This section contains a more detailed description of the optimal control model for bimanual reaching movements as presented in the main text, as well as a description of the implementation in Matlab (Mathworks [Natick, MA]). Furthermore, a sensitivity analysis of the correction rate in respect to arm stiffness is provided.

System Model

The model and its parameters are to a large degree derived from previous work on the two-degree-of-freedom arm [S1]. Because of the interactions between the elbow and shoulder joint, this system is nonlinear. Given the short length of the reaching movement (10 cm), I used a local linear approximation of the system in Euclidian coordinates. However, very similar results can be obtained with a nonlinear, joint-based model, by using iterative techniques to derive the optimal control policy [S2]. In the model, a state vector that contains 12 variables conceptualizes each hand. The state vector is composed of the x and y Euclidian coordinates of position (p), velocity (v), and force (f), muscle activation (h), and target position (g). The kinematics are modeled with

$$\begin{aligned} p_{t+1} &= p_t + \Delta t v_t \\ v_{t+1} &= v_t + \Delta t \frac{f_t}{I} \end{aligned}, \qquad \text{(S1)}$$

in which $\Delta t$is the size of the discrete time step, t is the number of time steps, and I is the inertia of the arm at the endpoint (0.5 kg).

I simulated the delay in the muscles by using two-coupled first-order low-pass filters, with time constants $\tau_1 = \tau_2 = 40$ ms [S3], that related the force (f) to the motor command (u). Thus, the produced force is a low-pass-filtered version of the motor command u. Again, the "muscle activations" are conceptualized in Eucledian coordinates because I am using a local linear approximation to a nonlinear system:

$$\begin{aligned} f_{t+1} &= f_t + \Delta t/\tau_2 (h_t - f_t) \\ h_{t+1} &= h_t + \Delta t/\tau_1 (u_t - h_t) \end{aligned}. \qquad \text{(S2)}$$

To simulate sensory delay, I expanded the state space with a series of four-coupled first-order filters for the sensed position, velocity and force:

$$x_{t+1}^{(j)} = x_t^{(j)} + \Delta t/\tau_s \left(x_t^{(j-1)} - x_t^{(j)} \right), \text{with } j = 1, \ldots, 4. \qquad \text{(S3)}$$

The current state of the system is $x^{(1)}$, and the state that can be sensed is $x^{(4)}$, in which x is used as a placeholder for p, v, or f. The time constant for each filter was $\tau_s = 15$ ms.

In summary, the state vector for each hand is

$$\mathbf{x}_t = \left[p_t^{(1)} \quad v_t^{(1)} \quad f_t^{(1)} \quad h_t \quad g \quad p_t^{(2)} \quad v_t^{(2)} \quad f_t^{(2)} \quad \ldots \quad p_t^{(4)} \quad v_t^{(4)} \quad f_t^{(4)} \right]^T. \qquad \text{(S4)}$$

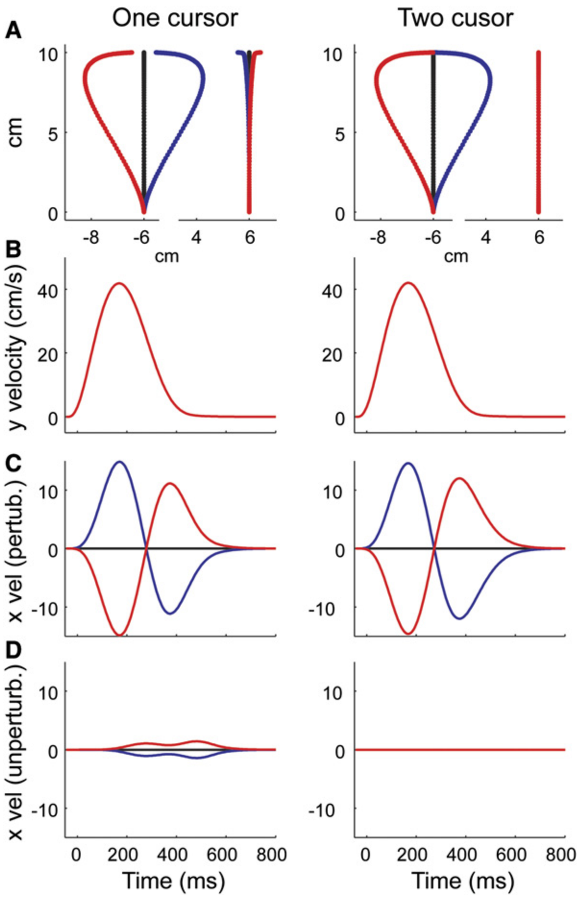

Figure S1. Optimal Control Theory Predictions for Bimanual Reaching Task

Predictions of optimal control theory for one-cursor (left) and two-cursor (right) conditions, if the left hand is perturbed with a leftward (red) or rightward (blue) directed force field. Position traces for left and right hand (A), forward (y) velocity of the perturbed hand (B), lateral (x) velocity of the perturbed hand (C), and lateral (x) velocity of the unperturbed hand (D) are shown.

And the sensed state is

$$\mathbf{y}_t = \left[p_t^{(4)} \quad v_t^{(4)} \quad f_t^{(4)} \right]. \qquad \text{(S5)}$$

By defining the appropriate matrices, the whole time-discrete system can be written as follows:

$$\begin{aligned} \begin{bmatrix} \mathbf{x}_L \\ \mathbf{x}_R \end{bmatrix}_{t+1} &= \mathbf{A} \begin{bmatrix} \mathbf{x}_L \\ \mathbf{x}_R \end{bmatrix}_t + \mathbf{B} \begin{bmatrix} \mathbf{u}_L \\ \mathbf{u}_R \end{bmatrix} \\ \begin{bmatrix} \mathbf{y}_L \\ \mathbf{y}_R \end{bmatrix}_{t+1} &= \mathbf{H} \begin{bmatrix} \mathbf{x}_L \\ \mathbf{x}_R \end{bmatrix}_t \end{aligned}. \qquad \text{(S6)}$$

It is important to note that A, B, and H are block diagonal; i.e., the left hand has no influence on the right hand and vice versa.

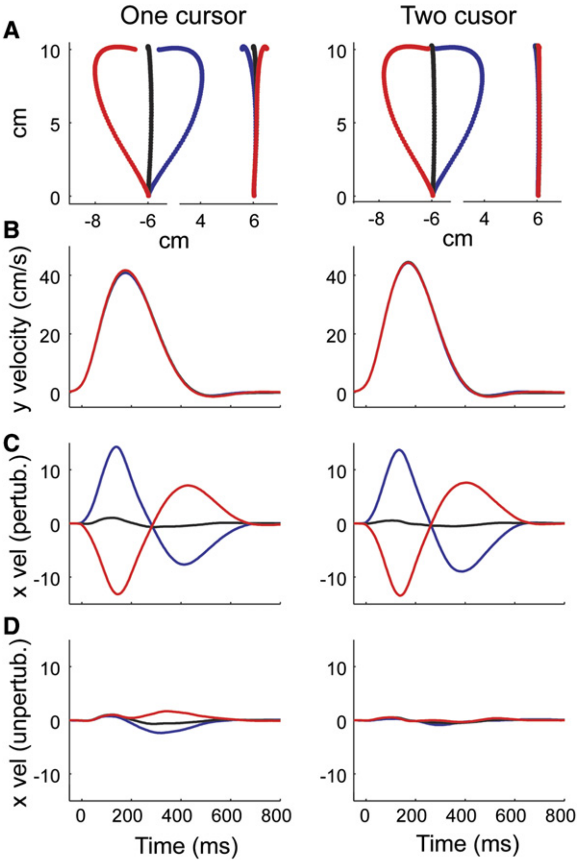

Figure S2. Experimental Data for Bimanual Reaching Task

Experimental data from experiment 1, plotted in the same format as in Figure S1. Results are averaged across hands and participant and displayed such that the perturbed hand appears on the left. As the arm stiffness of the perturbed hand increases, the within-hand correction rate increases and the between-hand correction decreases.

The stiffness of the perturbed arm was the only system parameter that was fitted to the experimental data. I used the data from the two-cursor condition and minimized the squared difference between the observed and simulated perturbation. The estimated stiffness term was 129.9 N/m.

The system matrices can be generated by calling M = bimoc_system('system').

The Cost Functions

The cost function for the two-cursor condition is composed of three terms:

$$J = \sum_{t=1}^{T} w_{p,t}\left(\|p_{L,t} - g_L\|^2 + \|p_{R,t} - g_R\|^2\right) + w_{v,t}\left(v_{L,t}^2 + v_{R,t}^2\right) + w_u\left(u_{L,t}^2 + u_{R,t}^2\right). \tag{S7}$$

The third term is the time-independent control cost. The first term reflects the cost of not being at the target location, and the second term reflects the cost of not having zero velocity. These costs increase exponentially over the course of the movement up to the maximal movement time (900 ms), reflecting the requirement of being at the target with zero velocity in the end of the movement:

$$\begin{aligned} w_{p,t} &= c_p \exp\left(-\frac{(MT - t\Delta t)}{\tau_{\text{cost}}}\right) \\ w_{v,t} &= c_v \exp\left(-\frac{(MT - t\Delta t)}{\tau_{\text{cost}}}\right) \end{aligned}. \tag{S8}$$

To derive the relative weights of the costs and the time constant of the exponential rise, I estimated these parameters to fit the velocity profile of unperturbed movements, resulting in τ_{cost} = 51 ms, c_p = 40 m^{-2}, c_v = 5 s^2/m^2, and w_u = 5*10e-5/s. For the one-cursor task, I used the same cost function, but replaced the position term with a common spatial term (Equation 2). The time-dependent cost function can be written in the form of

$$J = \sum_{t=1}^{T} \mathbf{x}_t^T \mathbf{Q}_t \mathbf{x}_t + \mathbf{u}_t^T \mathbf{R} \mathbf{u}_t, \tag{S9}$$

and these matrices are generated for the one- and two-cursor conditions by J = bimoc_system('costfunction', M, 'cursor',number_of_cursors).

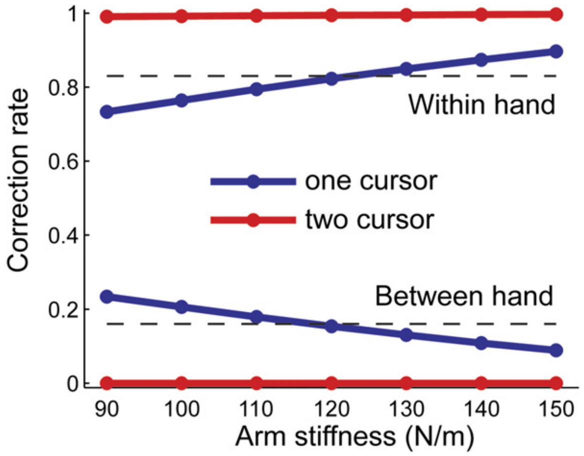

Figure S3. Sensitivity Analysis of the Correction Rate in Respect to the Parameter Arm Stiffness

Sensitivity analysis of the correction rate in respect to the parameter arm stiffness (N/m). As the arm stiffness of the perturbed hand increases, the within-hand correction rate increases and the between-hand correction decreases.

Derivation of the Optimal Control Policy

With the cost function, the Riccati Equations [S4] are used to calculated the optimal cost-to-go matrix $\mathbf{V}_t$and the optimal control gains $\mathbf{L}_t$. This computation is performed from the end to the start of the movement, iteratively moving backward in time:

$$\begin{aligned} \mathbf{V}_T &= \mathbf{Q}_T \\ \mathbf{L}_t &= \left(\mathbf{R} + \mathbf{B}^T \mathbf{V}_{t+1} \mathbf{B}\right)^{-1} \mathbf{B}^T \mathbf{V}_{t+1} \mathbf{A} \\ \mathbf{V}_t &= \mathbf{A}^T \mathbf{V}_{t+1} \mathbf{A} - \mathbf{A}^T \mathbf{V}_{t+1} \mathbf{B} \mathbf{L}_t + \mathbf{Q}_t \end{aligned}. \tag{S10}$$

The control gains allow us to compute the motor commands, given an estimate of the current state, $\mathbf{u}_t = -\mathbf{L}_t \widehat{\mathbf{x}}_t$, that minimize the cost function of the one-cursor and two-cursor condition, respectively. The main prediction of optimal control theory, independent

control for the two-cursor condition, and dependent control in the one-cursor condition arises from the fact that for the two-cursor cost function, all $\mathbf{L}_t$ are block-diagonal matrices, and for the one-cursor task, all $\mathbf{L}_t$ have nonzero off-diagonal entries. The derivation of the optimal control policy for a given system M and a cost function J is obtained by V = oc_solveLQG(M,J).

Force-Field Simulations

I then simulated the reaction of the system under the optimal control policy while a force field like in experiment 1 was applied to the arm. Each time step of the simulations consisted of the following:

1. Computation of the sensory inflow based on current state, $\mathbf{y}_t = \mathbf{H}\mathbf{x}_t$.
2. Measurement update of the state estimate $\widehat{\mathbf{x}}_t$given the sensory data $\mathbf{y}_t$with the optimal Kalman gains. The standard deviation of the measurement noise was 0.02 m for position, 0.2m/s for velocity, and 1 N for force, relative to the state noise with a standard deviation 1 [S3].
3. Calculation of the current control signal, with $\mathbf{u}_t = -\mathbf{L}_t\widehat{\mathbf{x}}_t$.
4. Simulation of the next time step of the real system with the system equations (Equation S6). For the first simulation, I used the system as described above. For another, I applied a leftward-pointing force field to the left hand, and for a last simulation, a rightward-pointing force field.
5. Time update of the Kalman filter to update the state estimate assuming that there is no force field applied to the hand. This step is the forward model prediction of the controller.

These steps are implemented in the function x = oc_runsystem(M,V,J);.

No noise was added to the motor output, and sensory noise was assumed to be of the same size as used in [S3]. Figure S1 shows the prediction of the model under the one- and two-object conditions. Figure S2 shows the mean experimental data in the same format, averaged over participants and hands. These figures can be regenerated with bimoc_simulate('simulate_plot') and bimoc_simulate('data_plot');

Sensitivity Analysis of Arm Stiffness

Most model parameters could be either derived from previous modeling work or fitted from the unperturbed movement. Only the stiffness of the perturbed arm, i.e., the mechanical resistance of the arm to the force field, had to be estimated by the adjustment of this parameter so that the squared differences between predicted and observed x velocity of the perturbed hand could be minimized (Figures S1C and S2C). This resulted in an estimate of 129.9 N/m. The sensitivity of the predicted within- and between-hand correction rate depending on the value of the arm stiffness is displayed in Figure S3. As the arm stiffness of the perturbed hand increases, the within-hand correction rate increases and the between-hand correction decreases because more of the correction is performed by the mechanical resistance of the arm against perturbations. Thus, the exact sharing of correction between the arms depends on the stiffness parameter, which for our setup could not be derived with high confidence. Despite this limitation, the qualitative prediction of there being no sharing of the correction in the two-cursor condition and shared corrections in the one-cursor condition (in the correct order of magnitude) is stable across a wide range of parameters and model specifications.

Supplemental References

S1. Shadmehr, R., and Mussa-Ivaldi, F.A. (1994). Adaptive representation of dynamics during learning of a motor task. J. Neurosci. *14*, 3208–3224.

S2. Todorov, E., and Li, W. (2005). A generalized iterative LQG method for locally-optimal feedback control on constrained nonlinear stochastic systems. Proceedings of the American Control Conference.

S3. Todorov, E. (2005). Stochastic optimal control and estimation methods adapted to the noise characteristics of the sensory motor system. Neural Comput. *17*, 1084–1108.

S4. Todorov, E. (2007). Optimal control theory. In Bayesian Brain. Probabilistic Approaches to Neural Coding, K. Doya, S. Ishii, A. Pouget, and R.P.N. Rao, eds. (Boston: MIT Press), pp. 269–298.
